# Community Assembly Mechanism of Woody Plants at Different Successional Stages in Karst Areas Based on Functional Traits and Phylogeny

**DOI:** 10.1002/ece3.73661

**Published:** 2026-05-18

**Authors:** Hongfen Hu, Mingtai An, Kun Wang, Li Tian, Feng Liu, Yiran Wang

**Affiliations:** ^1^ School of Ecology and Nature Conservation Beijing Forestry University Beijing China; ^2^ College of Forestry Guizhou University Guiyang China; ^3^ Research Center of Biodiversity and Nature Conservation Guizhou University Guiyang China; ^4^ State‐Owned Longli Forest Farm of Guizhou Province Longli China; ^5^ Key Laboratory of Plant Resource Conservation and Germplasm Innovation in Mountainous Region (Ministry of Education), College of Life Sciences/Institute of Agro‐bioengineering Guizhou University Guiyang China; ^6^ Research Center of Karst Ecological Environment, Guizhou University Guiyang China

**Keywords:** community assembly mechanism, functional traits, karst, phylogeny, succession

## Abstract

The mechanism of community assembly is a fundamental aspect of community ecology. In this study, the community assembly mechanism in karst areas was examined by investigating woody plants in 28 30 m × 30 m plots across seven distinct stages of succession in Maolan National Nature Reserve (Guizhou Province, southwestern China), a representative and well‐preserved subtropical karst forest system. The findings indicated that (1) Phylogenetic signals (Blomberg's *K*) for most functional traits followed a unimodal pattern, being suppressed in early stages but peaking in Stage III (*K* > 1) as *Anacardiaceae*‐dominated communities transitioned toward *Lauraceae*‐dominated stands. (2) The mean pairwise trait distance (traitMPD) and mean nearest trait distance (traitMNTD), along with the nearest taxon index (NTI), shifted from overdispersion (Stages I–III) to clustering (Stages IV–VII), reflecting that limiting similarity is eventually overridden by environmental filtering as lithological stress intensifies, while the net relatedness index (NRI) showed no significant variation. (3) Hierarchical partitioning confirmed that environmental distance (80.54%) exerted a much stronger influence than geographical distance (9.95%), collectively explaining 87.9% of community variation. We conclude that niche‐based processes remain the primary driver of karst forest succession. In early succession, where overdispersion indicates dominant competition, restoration should prioritize nitrogen‐fixing and deciduous pioneers to accelerate nutrient sequestration. Conversely, late‐stage clustering necessitates selecting phylogenetically distinct, stress‐tolerant clades to enhance drought insurance and navigate the intensified environmental filtering of maturing karst forests.

## Graphical abstract

This study reveals a shift in woody plant community assembly from competitive exclusion in early succession to habitat filtering in later stages within karst areas. Environmental distance (80.54%) contributed significantly more than geographical distance (9.95%), providing strong quantitative evidence that niche processes, particularly habitat filtering, dominate community assembly in these ecologically fragile regions.

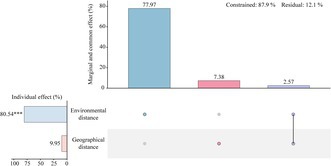

## 摘要

群落构建机制是群落生态学研究的核心内容。本文以贵州茂兰国家级自然保护区(中国西南部典型的亚热带喀斯特森林)为研究对象，通过调查7个不同演替阶段28个30 m × 30 m样方内的木本植物，探讨了喀斯特地区不同演替阶段的群落构建机制。结果表明:(1)大多数功能性状的系统发育信号(Blomberg's *K*)呈单峰趋势，在演替初期受到抑制，且在阶段III(*K* > 1)达到峰值，此时群落正由漆树科(*Anacardiaceae*)主导向樟科(*Lauraceae*)主导过渡。(2)平均成对性状距离(traitMPD)、平均最近相邻性状距离(traitMNTD)以及最近亲缘关系指数(NTI)均从演替初期的发散(阶段I–III)转变为后期的聚集(阶段IV–VII)；这反映出随着岩溶胁迫的加剧，生境过滤最终取代了生物间竞争排斥成为群落构建的主导力量，而净亲缘关系指数(NRI)未表现出显著变化。(3)层次分割分析证实，环境距离的解释率(80.54%)远高于地理距离(9.95%)，两者共同解释了87.9%的群落变化，进一步验证了生境过滤的主导作用。总体而言，本研究认为基于生态位的确定性过程是驱动喀斯特森林演替的主要动力。在演替初期，群落表现为发散结构，生物间竞争排斥作用主导群落构建，恢复工作应优先选择具固氮能力的树种和落叶树种以加速养分累积；而在演替后期，由于生境过滤加剧导致群落聚集，则需选择系统发育关系较远且具较强逆境适应性的类群，以增强群落的“干旱适应”能力并应对成熟喀斯特森林严苛的生境。

## 1 Introduction

The succession of a community is the process of gradual replacement, renewal and stability of species in the community, that is, the dynamic process of community assembly (Ma et al. 2022). Karst ecosystems represent a typical extreme habitat, characterized by severe surface fragmentation, shallow and discontinuous soils, and low water‐holding capacity, which jointly impose strong abiotic constraints on plant establishment and persistence (Li et al. 2026). For ecologically fragile karst areas, it is extremely important to determine the assembly process of plant communities to conserve soil and water as well as to prevent vegetation degradation. Due to rapid vegetation change and the wide range of rocky desertification in karst areas, it is increasingly important to study the community assembly mechanism during karst succession (Hu et al. 2017). Although successional communities are considered ideal for testing the mechanism of community assembly, research on the mechanism of community assembly based on niche theory and neutral theory has gradually increased in recent years (Han et al. 2023; Martínez‐Ramos et al. 2021). Compared with that in non‐karst areas, research on the community assembly mechanism at different successional stages in karst areas remains insufficient.

The two primary theories explaining the mechanism of community assembly are niche theory and neutral theory (He et al. 2022). Niche theory posits that environmental filtering and competitive exclusion among organisms are pivotal factors in community assembly (Tilman 1982; Kunstler et al. 2012). Within this framework, rigorous environmental filtering in karst ecosystems is expected to partition species based on adaptive functional traits, potentially yielding predictable and non‐random phylogenetic structures in instances where such traits are phylogenetically conserved (Zhou et al. 2026). When functional traits are phylogenetically conserved, environmental filtering tends to produce phylogenetic clustering, whereas trait convergence or competitive interactions may lead to phylogenetic overdispersion or random patterns (Webb et al. 2003). In contrast, neutral theory posits that random processes such as drift and dispersal are the primary drivers of community assembly, resulting in the random distribution of functional traits and a lack of phylogenetic structure within the community (Hubbell 2001). However, most ecologists conclude that niche theory and neutral theory are not mutually exclusive in relation to community assembly, and the central point of contention between these theories lies in determining the respective contributions of niche processes and neutral processes to community assembly (Leibold and McPeek 2006). In the study of different successional stages, the classic theory of community assembly holds that succession is controlled by niche processes. In the early stage of succession, environmental filtering determines community assembly, and with the progression of succession, competitive exclusion among organisms may surpass environmental filtering to become the determining factor of community assembly (Poorter et al. 2004).

In early studies of the mechanism of community assembly at different successional stages, ecologists primarily investigated the mechanism of community assembly at the species level based on taxonomic composition or species diversity (Doxa et al. 2020). However, the community assembly at various stages of succession is a highly intricate and dynamic process, and an exclusive focus on species changes does not provide a comprehensive explanation of the assembly mechanism. In contrast to the constraints imposed by species on the dynamics of community succession, functional traits represent the capacity of species to adapt to the environments of different successional stages during community formation, thereby effectively conveying the ability of plants to acclimate to external conditions (Wang, Song, et al. 2021; Wang, Wang, et al. 2021). Phylogeny encompasses evolutionary data among species and can elucidate the impact of historical processes on community assembly (Du et al. 2022). The phylogenetic signals of functional traits serve as indicators of the correlation between phylogenetic and functional traits. Traits evolve conservatively when phylogenetic signals are significant, whereas traits evolve convergently when phylogenetic signals are not significant. Consequently, the examination of phylogenetic signals that are related to functional traits serves as the foundation for determining the mechanism of community assembly at different successional stages (Krishna et al. 2021). Environmental and geographical distances are crucial metrics for gauging habitat filtering and dispersal limitation, respectively, and can be used to quantify the involvement of niche and neutral processes in community assembly at different successional stages (Francisco et al. 2021). Consequently, a comprehensive understanding of community assembly mechanisms can be achieved by examining the phylogenetic signals of functional traits, dynamic changes in community functional traits and phylogenetic structures, and the contributions of environmental and geographical distances during succession.

Karst landforms constitute approximately 12% to 25% of Earth's land area and exhibit notable characteristics such as extensive surface fragmentation, shallow soil, limited water retention, and a diverse range of species (Ford and Williams 2007). Located in the southern region of Guizhou Province, the Maolan National Nature Reserve is recognized as Earth's largest and most primitive karst forest within the same latitudinal zone, so it is an ideal area for investigating the community assembly mechanism of karst mountains. Against this background, karst successional stages can be conceptualized as a gradient of fluctuating environmental filtering intensity, where shifting habitat conditions systematically modulate functional trait composition and subsequently reorganize phylogenetic structure. This framework establishes a mechanistic link from extreme habitat constraints to trait‐based filtering and, ultimately, to the emergence of phylogenetic patterns, providing a robust pathway for deciphering community assembly (Liu et al. 2024). As a gradient effect involving multiple environmental factors, it is more important to explore the community assembly mechanisms at different successional stages in different vegetation types based on functional traits and phylogeny (Martínez‐Ramos et al. 2021). Many ecologists have carried out studies to verify the community assembly mechanisms at different successional stages in different vegetation types. For example, in the early and middle stages of subalpine meadow succession, the process of plant community assembly is influenced mainly by environmental filtering, and in the later stages, it is influenced by a combination of competitive exclusion and neutral processes (Liu et al. 2021). In abandoned farmland, the dominant factor in the assembly of successional communities changes from environmental filtering to competitive exclusion among organisms (Csecserits et al. 2021). Although the above research results explain the research on zonal communities well, compared with those in non‐karst areas, research on the community assembly of different successional stages in karst areas remains limited.

To investigate the assembly mechanism of karst communities based on functional traits and phylogenetic analysis, woody plant communities at various successional stages in the Maolan karst area were studied. Within this framework, we selected 13 functional traits that capture major axes of plant ecological strategies (spanning vegetative growth, resource conservation, and regenerative cycles) and distinguished seven successional stages to represent a continuous gradient of vegetation recovery and environmental filtering intensity, thereby enabling a process‐based test of the proposed “habitat‐trait‐phylogeny” linkage. The space‐for‐time substitution (SFT) approach was applied, and the following questions were addressed: (1) Is there phylogenetic conservation of functional traits among woody plants in karst areas? In other words, are the functional traits significantly influenced by phylogeny? (2) How do the assembly mechanisms of woody plant communities change dynamically based on their functional traits and phylogeny at various successional stages in karst areas? (3) How do environmental and geographical distances impact the community assembly mechanism of woody plants in karst areas? By answering these questions, it is anticipated that the assembly mechanism of karst communities will be revealed, thereby providing a scientific basis for vegetation restoration and community reconstruction in karst areas.

## 2 Materials and Methods

### 2.1 Study Area

The plots are located in the Guizhou Maolan National Nature Reserve (107°52′10″–108°05′40″ E, 25°09′20″–25°20′50″ N), with an average elevation of 758.5 m. The region is characterized by a typical subtropical humid monsoon climate, with an average annual temperature of 15.3°C, a mean temperature of 5.2°C during the coldest month and 23.5°C during the hottest month, an annual precipitation amount of 1752.5 mm, and an average annual humidity of 83%. The study region exhibits a highly typical karst topography with an exposed rock outcrop rate exceeding 90%. The soil is predominantly black calcareous soil, characterized by high spatial heterogeneity, low alkalinity, rich organic matter content and other nutrients and has a shallow and discontinuous soil layer (Wu et al. 2021). The primary vegetation type is the subtropical karst evergreen and deciduous broad‐leaved mixed forest, dominated by species such as *Carpinus pubescens*, *Acer wangchii*, *Platycarya longipes*, *Boniodendron minus*, *Phoebe crassipedicella*, *Clausena dunniana*, and *Lindera communis*.

### 2.2 Plot Survey

**FIGURE 1:**
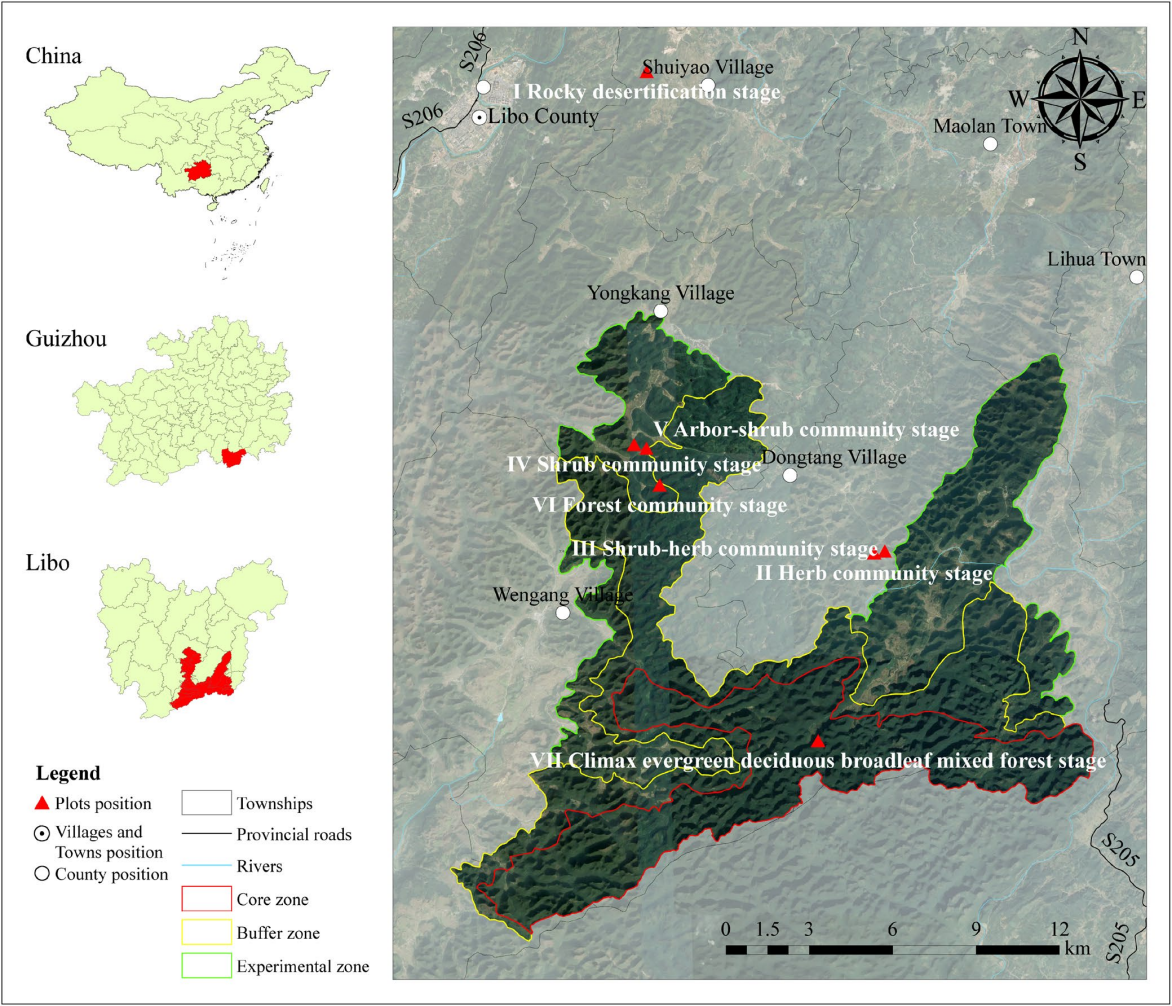
Map of the study area.

Succession is a protracted process, and long‐term monitoring in stationary plots is a desirable research approach (Lucas‐Borja and Delgado‐Baquerizo 2019). Given that succession in karst areas requires a minimum of one hundred years (Zeng et al. 2023), this investigation utilized the SFT approach. To isolate the effects of successional age from environmental noise, we implemented a stratified matching design to select 28 plots (30 m × 30 m; 4 per stage) across seven successional stages within and adjacent to the Maolan National Nature Reserve. These stages were delineated based on diagnostic indicators including rock exposure rate, vegetation coverage, plant height, and species composition (Table 1; Figure 1): rocky desertification stage (Stage I), herb community stage (Stage II), shrub‐herb community stage (Stage III), shrub community stage (Stage IV), arbor‐shrub community stage (Stage V), forest community stage (Stage VI), and climax evergreen deciduous broad‐leaved mixed forest stage (Stage VII).

**TABLE 1:** Summary of plots at different successional stages in the Maolan karst area.

Successional stage	Altitude (m)	Slope (°)	Slope position	Slope aspect	Vegetation coverage (%)	Community height (m)	Micro‐gully	Micro‐rock	Micro‐soil	Community appearance
I	830	30	Middle	WS	≤ 40	0.3–1	0.255	0.298	0.448	Rocky desertification
II	840	45	Middle	WN	≥ 70	0.5–1.5	0.522	0.027	0.451	Herb community
III	820	30	Middle	WS	≥ 85	1–2.5	0.090	0.021	0.890	Shrub‐herb community
IV	820	35	Middle	WS	≥ 85	2–4.5	0.368	0.483	0.149	Shrub community
V	820	35	Middle	WS	≥ 85	5–13	0.342	0.528	0.130	Arbor‐shrub community
VI	840	30	Middle	WS	≥ 85	12–18	0.227	0.368	0.405	Forest community
VII	850	30	Middle	WS	≥ 85	15–25	0.388	0.333	0.280	Climax evergreen deciduous broadleaf mixed forest

To ensure that community shifts were driven by successional chronosequence rather than spatial heterogeneity, we strictly matched topographic factors including soil type, altitude, slope, slope position, and slope aspect to select and establish 28 plots across the stages. Subsequent to plot establishment, micro‐topographic heterogeneity was quantified by partitioning each plot into 900 subplots (1 m × 1 m). Root microhabitats were surveyed and categorized into micro‐rock, micro‐gully, and micro‐soil based on specific bedrock exposure and soil depth thresholds (Table 1) (Zhu 1993).

**FIGURE 2:**
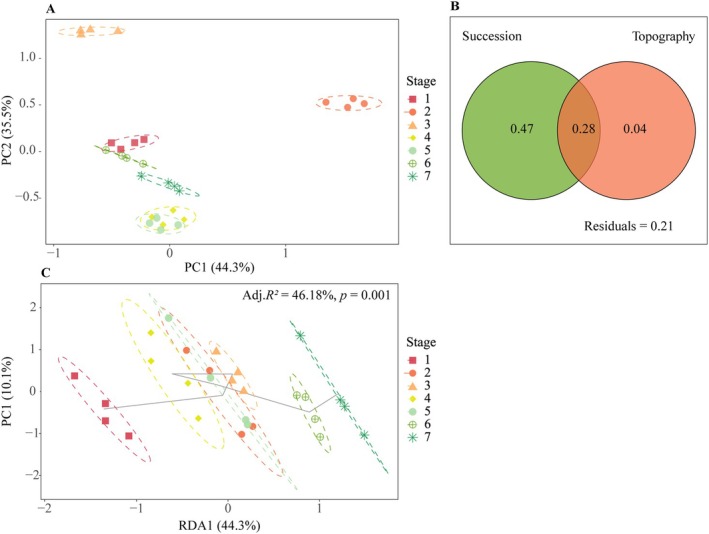
Validation of the space‐for‐time substitution by accounting for topographic heterogeneity. (a) PCA of topographic covariates. PC1 and PC2 explain 44.3% and 35.5% of the total topographic variation, respectively. (b) Variation partitioning of community structure. Values represent the pure effect of succession (0.47), the pure effect of topography (0.04), and their shared effect (0.28). (c) Partial RDA ordination. The trajectory shows significant community shifts along the successional gradient after controlling for topography (Adj. *R* ^2^ = 46.18%, *p* = 0.001).

To validate the SFT substitution and account for pre‐existing spatial gradients, topographic covariates were synthesized via principal component analysis (PCA) (Legendre and Legendre 2012) and incorporated as conditioning variables in variation partitioning analysis (VPA) (Borcard et al. 1992) and partial redundancy analysis (pRDA) (Legendre and Legendre 2012) to statistically isolate the pure effect of successional age. VPA revealed that successional age independently accounted for 47.0% of the total variation, whereas the pure effect of topographic heterogeneity was minimal at 4.4%. pRDA further confirmed that the successional effect remained highly significant (*F* = 57.19, *p* = 0.001) after statistically controlling for topographic covariates, while low variance inflation factors (VIF < 1.5) ensured the absence of multicollinearity among predictors. These results demonstrate that the successional chronosequence was successfully isolated from topographic interference (Figure 2).

### 2.3 Vegetation Survey

All woody individuals, including large woody vines, with diameters at breast height (DBH) ≥ 1 cm were examined within each 30 m × 30 m plot. The species names and number of trees were recorded. At the same time, the DBH was measured by a DBH ruler, and the heights were measured by an Haguang Direct Reading Tree Altimeter CGQ‐1.

The importance value (IV) was calculated as follows:

$$\mathrm{IV}=\frac{\mathrm{RF}+\mathrm{RD}+\mathrm{RDo}}{3}$$

where RF, RD, and RDo represent relative frequency, relative density, and relative dominance, respectively. Relative dominance was calculated based on basal area at breast height for tree layer species and basal area at stem base for shrub layer species (Curtis and McIntosh 1951).

### 2.4 Construction of a Functional Trait Tree and Phylogenetic Tree

In this study, based on survey data from Cornelissen's functional trait collection handbook (Cornelissen et al. 2003) and relevant literature and books, 13 key functional traits of plants were obtained. The parameters included the following: life form (LF), photosynthetic type (PT), nitrogen fixation type (NFT), maximum tree height (MTH), leaf lifespan (LL), leaf size (LS), leaf texture (LT), first flowering phase (FFLP), flowering phase (FLP), pollination mode (PM), first fruit stage (FFRP), fruiting phase (FRP), and seed dispersal mode (SDM) (Table 2). These traits collectively capture key ecological strategies (Pérez‐Harguindeguy et al. 2013). Specifically, PT, LL, LS, and LT reflect resource acquisition and conservation along the leaf economics spectrum (Wright et al. 2004); LF and MTH indicate growth and spatial competition (Westoby 1998); FFLP, FLP, PM, FFRP, FRP, and SDM relate to reproduction and dispersal capabilities (Cornelissen et al. 2003); and NFT relates to nutrient cycling and stress tolerance.

**TABLE 2:** Types of plant functional traits.

Plant functional traits	Type	Unit	Standard of measurement
Life form (LF)	Qualitative		(1) Vine, (2) Shrub, (3) Tree
Photosynthetic type (PT)	Qualitative		(1) C3, (2) C4
Nitrogen fixation type (NFT)	Qualitative		(1) Non‐nitrogen‐fixing, (2) Nitrogen‐fixing
Maximum tree height (MTH)	Quantitative	m	The average of the highest top 5% individuals of each species
Leaf lifespan (LL)	Qualitative		(1) Evergreen, (2) Deciduous
Leaf size (LS)	Qualitative	cm^2^	(1) ≤ 13.70 cm^2^, (2) 13.70–21.00 cm^2^, (3) 21.00–28.10 cm^2^, (4) 28.10–35.60 cm^2^, (5) 35.60–47.00 cm^2^, (6) 47.00–67.80 cm^2^, (7) ≥ 67.80 cm^2^
Leaf texture (LT)	Qualitative		(1) Membranous, (2) Paper, (3) Leathery
First flowering phase (FFLP)	Quantitative	month	Specific months of first flowering, 1–12
Flowering phase (FLP)	Quantitative	month	Lasting month from first to last flowering, 1–12
Pollination mode (PM)	Qualitative		(1) Insect pollination, (2) Wind pollination
First fruiting phase (FFRP)	quantitative	month	Specific months of initial results, 1–12
Fruiting phase (FRP)	Quantitative	month	Month of duration from first result to last result, 1–12
Seed dispersal mode (SDM)	Qualitative		(1) Zoochory, (2) Anemochory, (3) Autochory

LS was classified into seven ordinal categories based on its empirical quantile distribution, and the consistency between continuous and categorical representations was assessed using Gower distance (Gower 1971) and a Mantel test (*r* = 0.417, *p* = 0.001). The categorical LS showed no significant correlation with phylogenetic distance (Mantel 1967) (*r* = −0.016, *p* = 0.755), indicating that the classification did not distort trait‐space topology. A functional trait characteristic matrix was obtained by standardizing the functional trait data with a log_10_ logarithmic transformation, which was subsequently transformed into a Euclidean distance matrix. The resulting distance matrix was then subjected to hierarchical clustering to generate a functional trait tree (Petchey and Gaston 2002). Additionally, a list of families, genera, and species was generated based on the names of species at different successional stages in accordance with the Angiosperm Phylogeny Group IV (APG IV) classification, while phylogenetic trees were constructed based on the evolutionary divergence among species (Bremer et al. 2009).

### 2.5 Phylogenetic Signals of the Functional Traits

To examine whether phylogenetic signals vary across successional stages, we calculated Blomberg's *K* statistic (Blomberg et al. 2003) separately for each of the seven successional phases (Stages I–VII): *K* = 0 represents no phylogenetic signal, *K* = 1 indicates a strong phylogenetic signal, and the trait evolves according to Brownian motion. A weak phylogenetic signal is indicated by 0 < *K* < 1, whereas *K* > 1 indicates a very strong signal and that the trait values are more similar than those expected under Brownian motion. To assess the importance of phylogenetic signals in functional traits, a comparison was made between the observed *K* values and the null model. A significant phylogenetic signal for functional traits was inferred if the observed values exceeded 95% of the null model value (*p* < 0.05) (Webb et al. 2003).

### 2.6 Species, Functional and Phylogenetic α Diversity Indices

In this study, the species richness index (SR), Simpson diversity index (*λ*), and Pielou evenness index (Je) were selected for use as the traditional species *α* diversity indices. The calculation formulas are as follows:

Species richness index,SR=S

$$\text{Simpson diversity index},\lambda =1-\sum {p_{i}}^{2}$$

$$\text{Pielou evenness index},\mathrm{Je}=-\frac{\sum p_{i}\ln\left(p_{i}\right)}{\ln S}$$

In the formulas, *S* denotes the total number of species, and *p*_*i*_ denotes the relative abundance of the ith species (Qianwen et al. 2022).

The functional richness index (FRic), Rao's quadratic entropy index (RaoQ), and functional evenness index (FEve) were selected as the functional *α* diversity indices, which were calculated based on 13 functional traits that were standardized by a log_10_ logarithmic transformation. The calculation formulas are as follows:

$$\text{Functional richness index},\text{FRic}=\frac{{\mathrm{SF}}_{\mathrm{ci}}}{R_{c}}$$

$${\mathrm{Rao}}^{'}s\;\text{quadratic entropy index},\text{RaoQ}=\sum_{i=1}^{S-1}\sum_{i=i+1}^{S}d_{\mathrm{ij}}p_{i}p_{j}$$

$$\text{Functional evenness index},\text{FEve}=\frac{\sum_{i=1}^{S-1}\min\left({\mathrm{PEW}}_{i}\frac{1}{S-1}\right)-\frac{1}{S-1}}{1-\frac{1}{S-1}}$$

$${\mathrm{PEW}}_{i}=\frac{{\mathrm{EW}}_{i}}{\sum_{i=1}^{S-1}{\mathrm{EW}}_{i}}$$

$${\mathrm{EW}}_{i}=\frac{\text{dist}\left(i,j\right)}{w_{i}+w_{j}}$$

In the formulas, SF_ic_ denotes the niche of the species in the community, *R* denotes the absolute eigenvalue of trait c in the community, *S* denotes the total number of species, *d*_*ij*_ denotes the overlap of the probability density function of the trait values of species *i* and species *j*, *p*_*i*_ and *p*_*j*_ denote the proportions of the number of individuals of species *i* and species *j* to the total number of individuals of species in the community, EW denotes the evenness, dist (*i, j*) denotes the Euclidean distances of species *i* and *j*, and *w*_*i*_ denotes the number of species *i*.

Faith's phylogenetic diversity index (PD), the mean pairwise phylogenetic distance (MPD), and the mean nearest phylogenetic distance (MNTD) were used to measure the phylogenetic *α* diversity of the community. The calculation formulas are as follows:

$${\text{Faith}}^{‘}s\;\text{phylogenetic diversity index},\mathrm{PD}=B\times \frac{\sum_{i}^{B}L_{i}A_{i}}{\sum_{i}^{B}A_{i}}$$

$$\text{Mean pairwise phylogenetic distance},\mathrm{MPD}=\frac{\sum_{m}\sum_{n}d_{\mathrm{mn}}a_{m}a_{n}}{\sum_{m}\sum_{n}a_{m}a_{n}}$$

$$\text{Mean nearest phylogenetic distance},\text{MNTD}=\sum_{m}^{s}\min\left(d_{\mathrm{mn}}\right)a_{m}$$

In the formulas, *n* refers to the node‐based index, *B* refers to the number of branches in the tree, *L*_*i*_ refers to the length of branch *i*, *A*_*i*_ refers to the average abundance of species sharing branch *i*, *d*_*mn*_ refers to the phylogenetic distance between species *m* and species *n*, *a*_*m*_ refers to the abundance of species *m*, and *S* refers to the total number of species (De Pauw et al. 2021).

### 2.7 Functional and Phylogenetic Structure Indices

The mean pairwise trait distance (traitMPD) and mean nearest trait distance (traitMNTD) were used to assess the functional trait structure of the community, while the nearest relatedness index (NRI) and nearest taxon index (NTI) were used to evaluate the phylogenetic structure of the community (Webb et al. 2003). The calculation formulas are as follows:

$$\text{Mean pairwise trait distance},\text{traitMPD}=-1\times \frac{{\text{traitMPD}}_{\mathrm{obs}}-{\text{traitMPD}}_{\text{mean}}}{{\text{traitMPD}}_{\mathrm{sd}}}$$

$$\text{Mean nearest trait distance},\text{traitMNTD}=-1\times \frac{{\text{traitMNTD}}_{\mathrm{obs}}-\text{trait}{\text{MNTD}}_{\text{mean}}}{\text{trait}{\text{MNTD}}_{\mathrm{sd}}}$$

$$\mathrm{Net}\;\text{relatedness index},\mathrm{NRI}=-1\times \frac{{\mathrm{MPD}}_{\mathrm{obs}}-{\mathrm{MPD}}_{\text{mean}}}{{\mathrm{MPD}}_{\mathrm{sd}}}$$

$$\text{Nearest taxon index},\mathrm{NTI}=-1\times \frac{{\text{MNTD}}_{\mathrm{obs}}-{\text{MNTD}}_{\text{mean}}}{{\text{MNTD}}_{\mathrm{sd}}}$$

In the formulas, traitMPD_obs_, traitMNTD_obs_, MPD_obs_, and MNTD_obs_ represent the actual observed values. The traitMPD_mean_, traitMNTD_mean_, MPD_mean_, and MNTD_mean_ values are the average values obtained from 999 simulations using a null model. The traitMPD_sd_, traitMNTD_sd_, MPD_sd_, and MNTD_sd_ values represent the standard deviations of the 999 simulated values.

If both traitMPD and traitMNTD > 0, the functional trait structure is clustered; if traitMPD and traitMNTD < 0, the functional trait structure is overdispersed; if traitMPD and traitMNTD = 0, the functional trait structure is random. If NRI and NTI > 0, the phylogenetic structure is clustered; if NRI and NTI < 0, the phylogenetic structure is overdispersed; if NRI and NTI = 0, the phylogenetic structure is random.

### 2.8 Environmental Distance and Geographical Distance

In this study, environmental distance was calculated using soil factors. Soil samples were taken at the four corners and at the centers of the 30 m × 30 m plots at each stage. A total of 20 soil samples were obtained for each stage, resulting in 140 soil samples across seven stages. Following the sampling process, a total of 10 soil moisture and nitrogen factors, namely, soil water content, SWC (%), soil organic carbon, SOC (g/kg), total nitrogen, TN (g/kg), total phosphorus, TP (g/kg), alkaline hydrolysis nitrogen, AN (mg/kg), available phosphorus, AP (mg/kg), SOC:TN, SOC:TP, TN:TP, and SOC:TN:TP, were measured as the environmental factors of the karst area.

The soil moisture content was measured by the drying method. The soil samples were returned to the laboratory, and the fresh weight of each soil sample was determined. The dry weight was determined after drying in an oven at 105°C for 8 h. The soil organic carbon, total nitrogen, total phosphorus, alkaline hydrolysis nitrogen, and available phosphorus values were measured by the dichromate oxidation method (Pribyl 2010), the Kjeldahl method (Bremner and Mulvaney 1982), the molybdate colorimetry method after H_2_SO_4_–HClO_4_ digestion (Pansu and Gautheyrou 2007), the microdiffusion technique (Mou et al. 1993), and the molybdenum antimony colorimetric method (Bao 2000), respectively.

The geographical distances were calculated according to the geographical coordinates of the center points of the 28 quadrats in different successional stages. Distance‐based Moran's eigenvector maps (dbMEM) were used to obtain the feature vector, and the significant values were filtered to construct the spatial matrix to obtain the geographical distances.

### 2.9 Data Statistics and Analysis

In this study, the soil environmental factors, functional traits, and phylogenetic structure indices at different successional stages are expressed as the means ± standard deviations, and the *α* diversity indices are expressed as the median and extreme values.

For the *α* diversity index, functional trait variation, and phylogenetic structure index, the data that satisfied a normal distribution and homogeneity of variance were analyzed by one‐way analysis of variance (ANOVA) and then tested by the least significant difference (LSD) test. Data that did not meet the assumptions of normality and homogeneity of variance were analyzed using the Kruskal–Wallis test and then back‐tested by the Dunn–Bonferroni method.

The impacts of environmental factors and geographical coordinates on the community assembly mechanisms were quantified by calculating the explanatory power of environmental distance and geographical distance for the community functional trait structure indices (e.g., traitMPD and traitMNTD) and phylogenetic structure indices (e.g., NRI and NTI) (Blundo et al. 2016).

Variation partitioning (VP) based on redundancy analysis (RDA) was employed to assess the linear correlations among the functional traits and phylogenetic structure indices, as well as those between the environmental distances and geographical distances. Additionally, hierarchical partitioning (HP) was utilized to determine the significance of environmental distances and geographical distances as well as their individual impacts on functional traits and phylogenetic structure indices (Lai et al. 2022).

PCA, VP and pRDA for topographic effects were calculated using the vegan package (Oksanen et al. 2011). Gower distances and Mantel tests for leaf area grading were estimated with the cluster, vegan, and ape packages (Paradis and Schliep 2019; Mächler et al. 2012). Blomberg's *K* and *p* values, traitMPD, traitMNTD, the NRI and NTI were estimated with the picante package (Kembel et al. 2010). The species, functional traits and phylogenetic *α* diversity indices were calculated using the vegan, FD and picante packages, respectively (Laliberté and Legendre 2010). RDA, dbMEM, and HP were calculated using the rdacca.hp., adespatial, and UpSetVP packages, respectively (Lai et al. 2022; Conway et al. 2017; Guénard and Legendre 2022; Mendiburu 2017). Graphs were generated with the ggplot2 and adespatial packages (Wickham 2009). All aforementioned analyses and mapping procedures were performed with R version 3.6.3 and SPSS. The phylogenetic tree was constructed on the ChiPlot website (https://www.chiplot.online/).

## 3 Results

### 3.1 Differences in Soil Factors at Different Successional Stages

The soil factors exhibited significant heterogeneity across successional stages (Table 3). This pattern was primarily driven by the successional chronosequence rather than topographic gradients (Figure 2B,C). SWC, SOC, TN, TP, and AN were highest at Stage VII (Table 3) and were lowest at Stage I (Table 3). AP, SOC:TN, SOC:TP, TN:TP, or SOC:TN:TP remained stable with succession. Overall, soil nitrogen and water contents in the karst area increased gradually throughout the successional process. Such temporal shifts in soil nutrients and water content align well with the observed transitions in community diversity and phylogenetic structure.

**TABLE 3:** Soil environmental factors for the woody plant communities at different successional stages in the Maolan karst area.

Successional stage	Item	SWC/(%)	SOC/(g/kg)	TN/(g/kg)	TP/(g/kg)	AN/(mg/kg)	AP/(mg/kg)	SOC: TN	SOC: TP	TN: TP	SOC: TN:TP
I	Mean	20.20	53.29	2.186	0.215	183.5	4.212	24.79	239.5	9.766	119.9
	SD	2.70	12.15	0.633	0.038	39.15	0.053	2.253	13.63	1.321	35.75
II	Mean	31.60	113.0	3.969	0.328	276.2	5.357	28.93	334.9	11.61	101.7
	SD	3.20	58.44	2.153	0.155	81.12	3.629	1.044	27.01	1.293	41.12
III	Mean	25.10	67.34	2.711	0.290	233.3	2.586	24.68	222.0	8.970	89.93
	SD	2.80	25.39	1.002	0.073	71.87	1.592	0.608	38.34	1.444	26.18
IV	Mean	32.70	141.8	7.439	0.830	436.7	9.596	18.66	174.6	9.163	23.14
	SD	2.80	33.14	1.149	0.114	86.14	4.129	1.487	46.66	1.837	4.035
V	Mean	29.10	92.94	5.324	0.369	357.3	4.622	17.43	243.5	13.95	48.02
	SD	7.40	22.50	1.302	0.052	72.50	1.916	0.134	28.40	1.651	7.562
VI	Mean	31.40	84.96	4.820	0.613	358.3	3.878	17.72	134.3	7.556	29.74
	SD	4.50	31.29	1.731	0.125	89.35	2.48	0.446	25.65	1.34	7.075
VII	Mean	50.70	295.2	15.28	1.509	712.6	25.876	19.48	193.3	9.980	13.04
	SD	8.60	101.7	5.577	0.164	160.4	20.662	0.91	48.16	2.819	1.836

### 3.2 Species Compositions at Different Successional Stages

**FIGURE 3:**
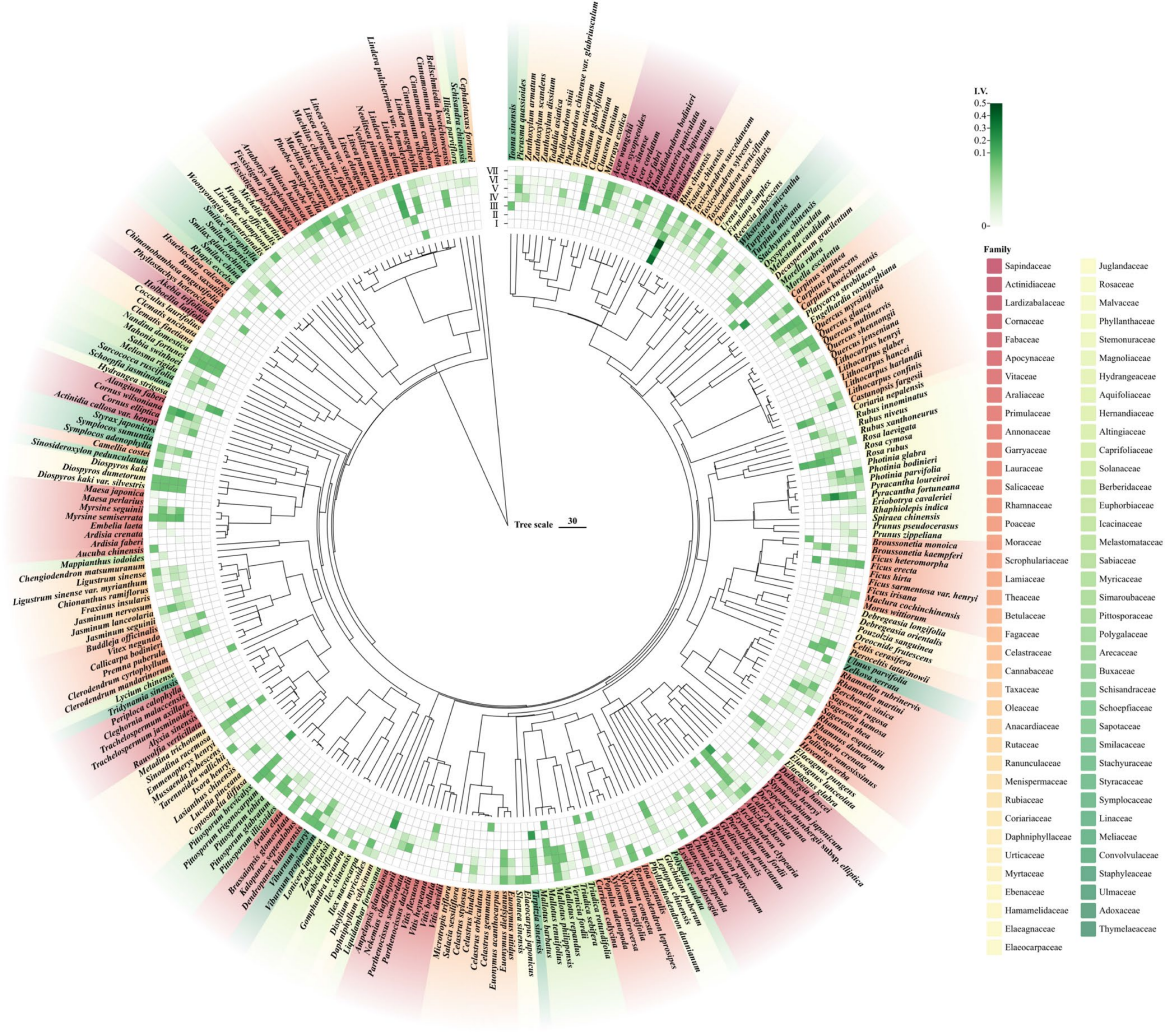
Phylogenetic tree and heatmap of the importance values for the woody plants at different successional stages in the Maolan karst area. The stages include the rocky desertification stage (Stage I), herb community stage (Stage II), shrub‐herb community stage (Stage III), shrub community stage (Stage IV), arbor‐shrub community stage (Stage V), forest community stage (Stage VI), and climax evergreen deciduous broad‐leaved mixed forest stage (Stage VII).

A total of 282 woody plant species from 187 genera and 75 families were recorded (Figure 3). Species richness increased across the chronosequence, with 15, 16, 30, 140, 141, 160, and 145 species identified from Stages I to VII, respectively. These shifts in taxonomic richness align with the observed transitions in community assembly patterns. Our analysis identified Anacardiaceae, Rosaceae, and Lauraceae as the most dominant families, accounting for 16.68%, 9.68%, and 8.96% of the total IV, respectively. Furthermore, Anacardiaceae dominated in the early successional stages (I–III, Figure 3), Rosaceae dominated the middle successional stages (III–V, Figure 3), and Lauraceae dominated the late successional stages (IV–VII, Figure 3). This replacement of dominant lineages mirrors the transition from overdispersion toward clustering in the community. *Albizia kalkora* was the only species present across all seven stages. Additionally, the codominant species of the first three stages (top five IVs) appeared only in the first five stages, and the codominant species of the last four stages appeared only in the last five stages.

### 3.3 Phylogenetic Signals of Functional Traits

**FIGURE 4:**
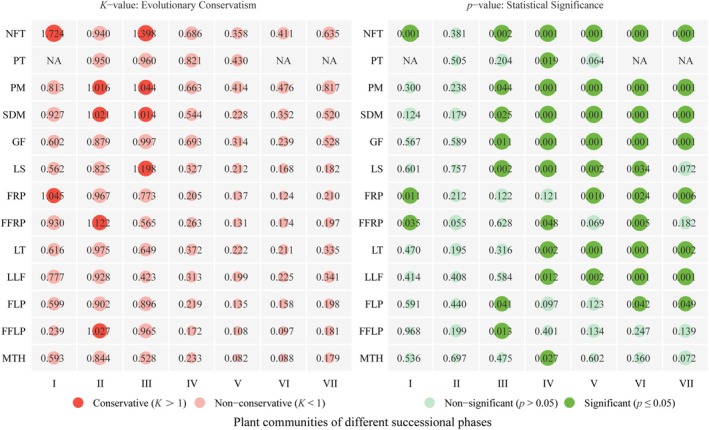
Phylogenetic signals of functional traits for the woody plant communities at different successional stages in the Maolan karst area. The stages include the rocky desertification stage (Stage I), herb community stage (Stage II), shrub‐herb community stage (Stage III), shrub community stage (Stage IV), arbor‐shrub community stage (Stage V), forest community stage (Stage VI), and climax evergreen deciduous broad‐leaved mixed forest stage (Stage VII). The functional traits include the nitrogen fixation type (NFT), photosynthetic type (PT), pollination mode (PM), seed dispersal mode (SDM), growth form (GF), leaf size (LS), fruiting phase (FRP), first fruit stage (FFRP), leaf texture (LT), leaf lifespan (LLF), flowering phase (FLP), first flowering phase (FFLP), and maximum tree height (MTH).

Phylogenetic signals across the seven successional stages revealed a dynamic temporal pattern (Figure 4). In Stages I–II, signals were generally weak or non‐significant, with NFT, FFRP, and FRP in Stage I as notable exceptions. The most pronounced signals occurred in Stage III, where the majority of traits reached statistical significance (*p* ≤ 0.05). Specifically, NFT, LS, SDM, and PM exhibited *K* > 1 during this stage, indicating strong evolutionary conservatism. This peak in K values aligns with the onset of substantial shifts in functional and phylogenetic structures. Subsequently, K values declined in Stage IV and continued to decrease in later stages (IV–VII), with MTH and LS losing significance by Stage VII (*p* > 0.05). In summary, trait conservatism peaked at mid‐succession and attenuated thereafter. This shift suggests that intensified environmental filtering or competition overrides evolutionary conservatism in shaping community assembly during later stages.

### 3.4 Taxonomic, Functional, and Phylogenetic *α* Diversity Values at Different Successional Stages

**FIGURE 5:**
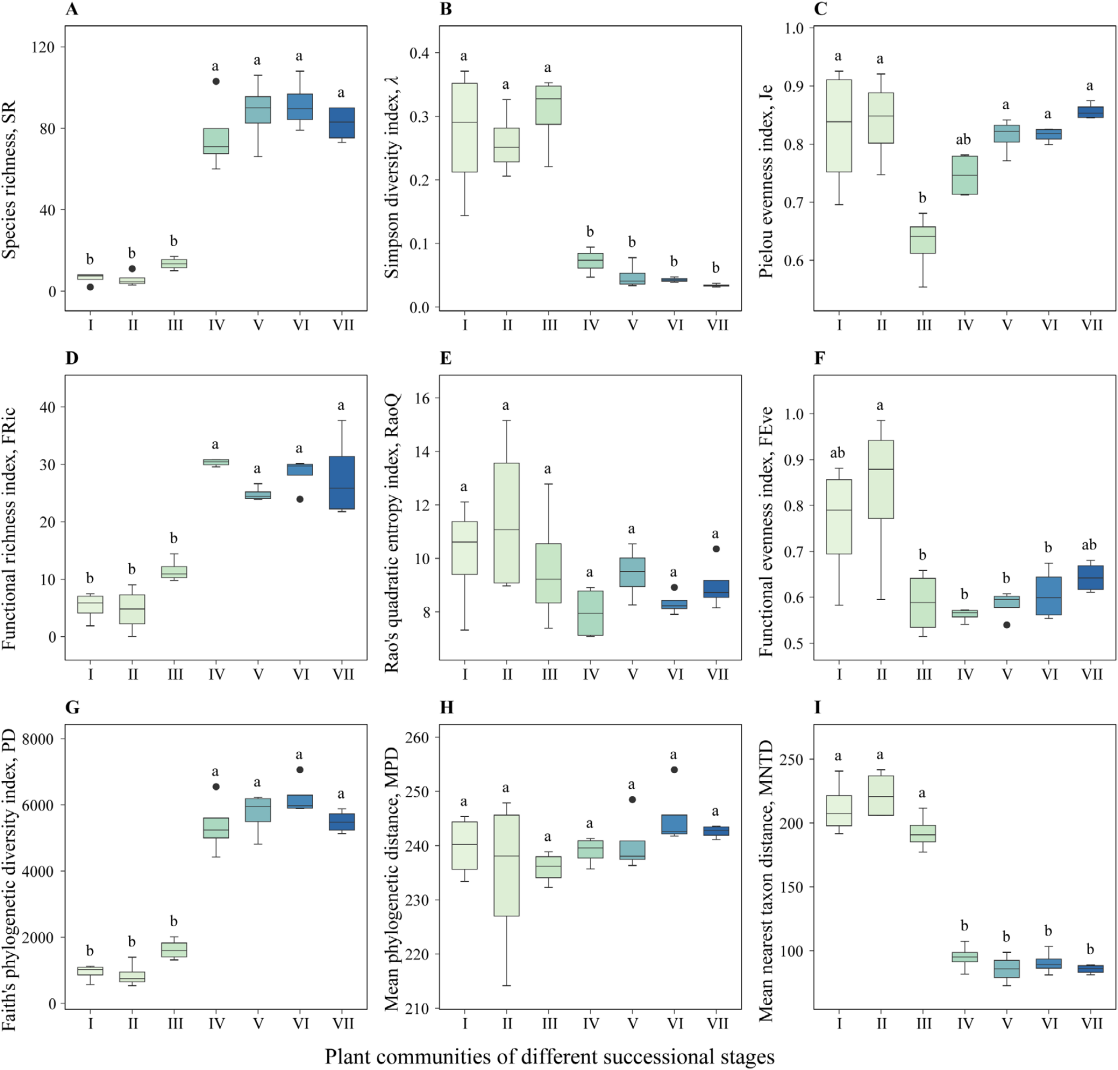
Taxonomic, functional, and phylogenetic *α* diversity values for the woody plant communities at different successional stages in the Maolan karst area; horizontal lines indicate median values, and different lowercase letters indicate significant differences (*p* < 0.05). The stages include the rocky desertification stage (Stage I), herb community stage (Stage II), shrub‐herb community stage (Stage III), shrub community stage (Stage IV), arbor‐shrub community stage (Stage V), forest community stage (Stage VI), and climax evergreen deciduous broad‐leaved mixed forest stage (Stage VII).

Except for RaoQ and MPD (Figure 5E,H), the taxonomic, functional, and phylogenetic *α* diversity indices of the woody plant communities in the Maolan karst area exhibited substantial changes at different successional stages (Figure 5). SR and PD increased initially and subsequently declined with succession, with the peak occurring in Stage VI (Figure 5A,G). The changes in FRic were similar, but the peak occurred in Stage VII (Figure 5D). Conversely, *λ* and MNTD were substantially greater in the first three successional stages than in the last four successional stages (Figure 5B,I). Notably, Stage III had the lowest *Je* values, which were substantially different from those of the other stages except Stage IV (Figure 5C). The highest values of the FEve were found in Stage II and differed considerably from those in Stages III–V (Figure 5F). These variations in *α* diversity coincide with the dynamic phylogenetic signals of traits such as NFT and LS, suggesting a linkage between trait conservatism and community diversity patterns.

### 3.5 Functional Trait Structure and Phylogenetic Structure at Different Successional Stages

**FIGURE 6:**
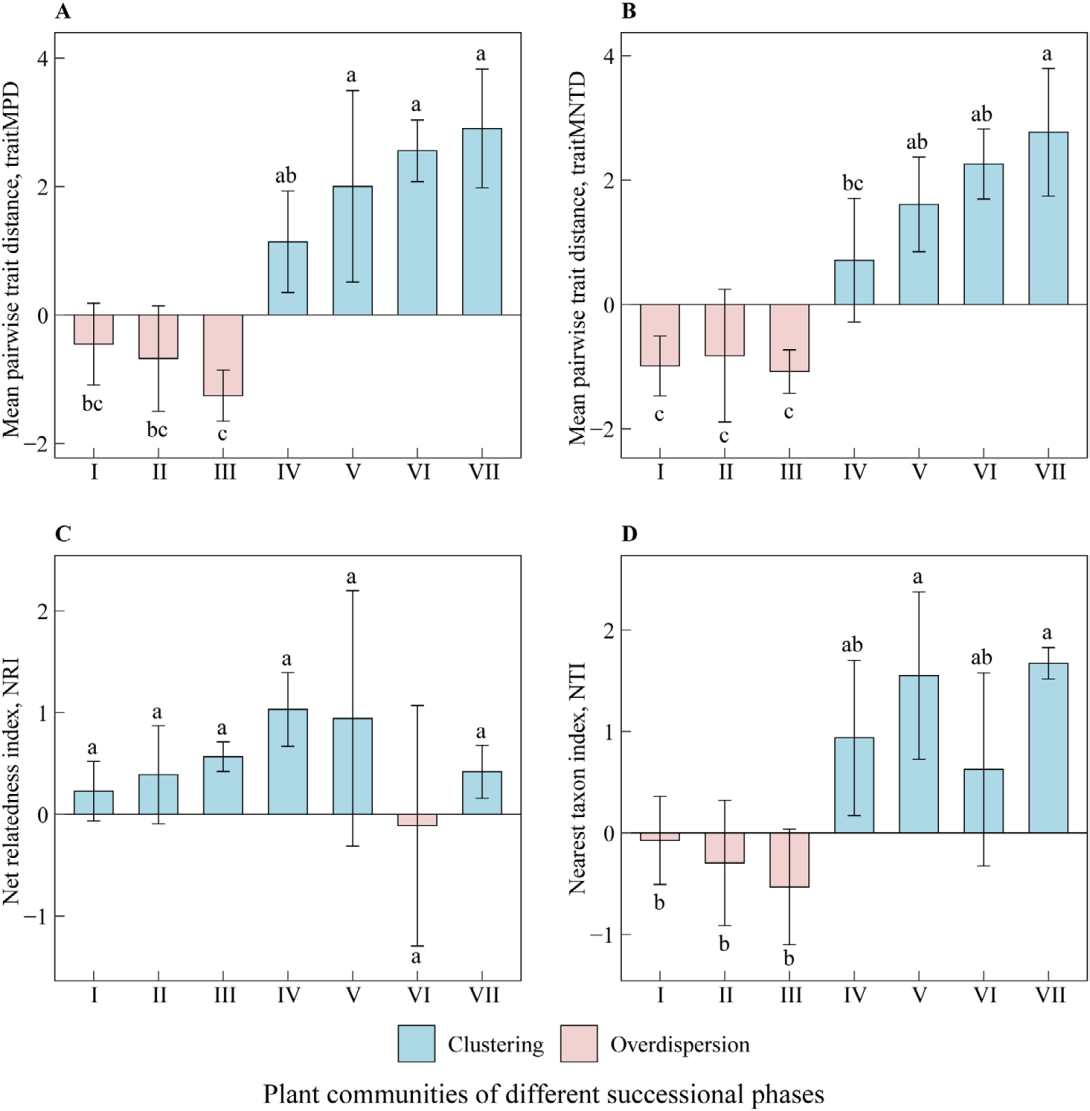
Functional and phylogenetic structures for the woody plant communities at different successional stages in the karst region (mean ± SD); different lowercase letters indicate significant differences (*p* < 0.05). The stages include the rocky desertification stage (Stage I), herb community stage (Stage II), shrub‐herb community stage (Stage III), shrub community stage (Stage IV), arbor‐shrub community stage (Stage V), forest community stage (Stage VI), and climax evergreen deciduous broad‐leaved mixed forest stage (Stage VII).

The dynamic changes in traitMPD, traitMNTD, NRI, and NTI across different successional stages in the Maolan karst area are shown in Figure 6. Except for NRI, the other three indices exhibit substantial differences across successional stages (Figure 6C). In Stages I–III, traitMPD, traitMNTD, and NTI were less than 0, indicating that the community structures exhibited overdispersion (Figure 6A,B,D). This overdispersion in early stages coincided with the presence of functional traits exhibiting significant phylogenetic signals (e.g., NFT and FFRP in Stage I). In Stages IV–VII, the traitMPD, traitMNTD, and NTI were all greater than 0, suggesting that the community structures tended toward clustering (Figure 6D). Notably, the transition from overdispersion to clustering in NTI and traitMNTD followed the peak of phylogenetic conservatism observed in Stage III. NRI values showed no significant variation across all successional stages (*p* > 0.05), although a non‐significant trend toward overdispersion was observed in Stage VI (Figure 6C). Collectively, these structural transitions align with the observed phylogenetic signals (e.g., NFT and FFRP in Stage I), indicating that functional and phylogenetic assembly follow interrelated but distinct trajectories.

### 3.6 The Impacts of Environmental and Geographical Distances on the Community Assembly Mechanism Across Varying Successional Stages

**FIGURE 7:**
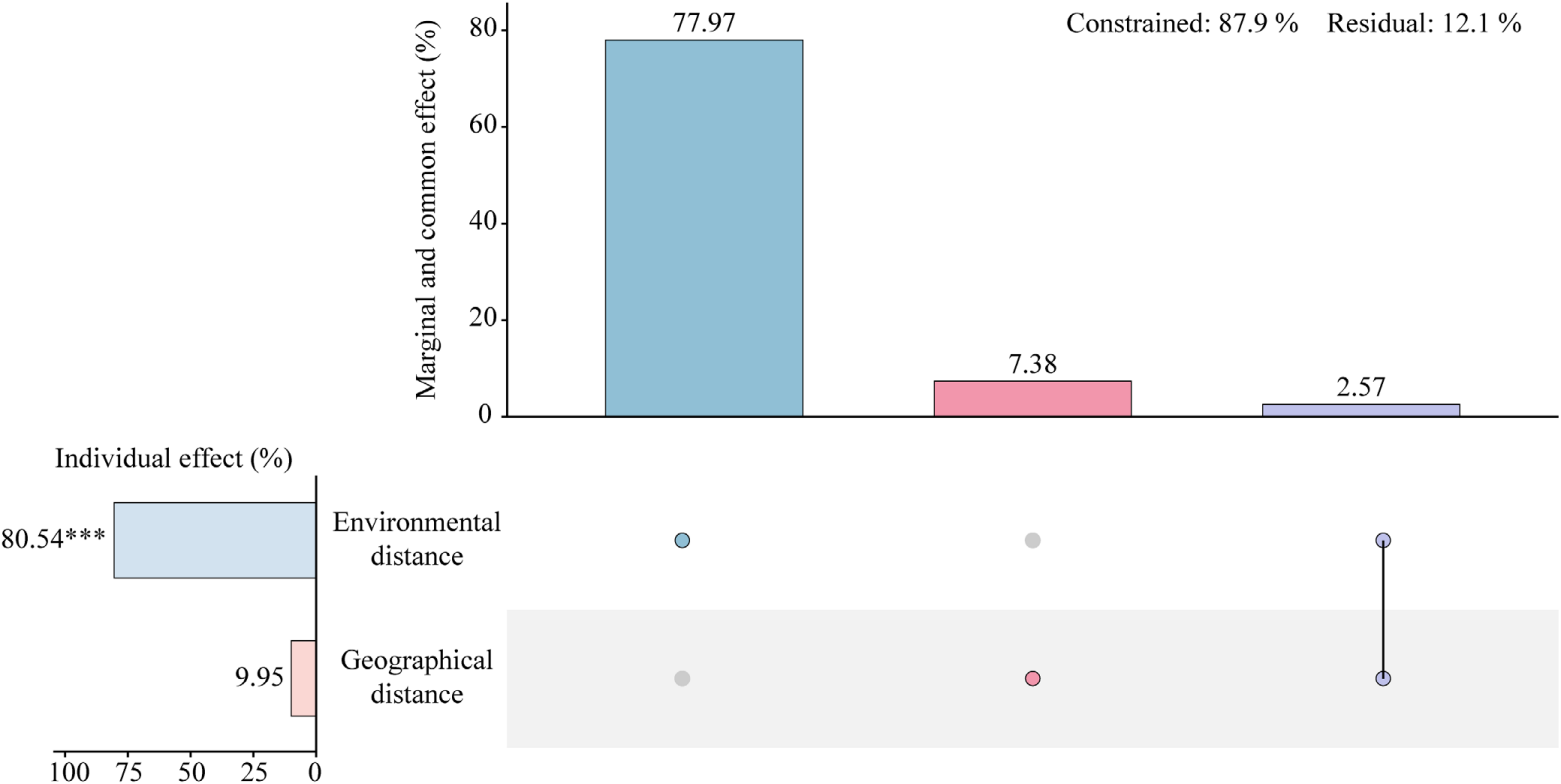
UpSetVP plot for the hierarchical and variation partitioning of community assembly. The bar plot on the left shows the individual effects of environmental distance (***, *p* < 0.001) and geographical distance (*p* > 0.05). The top bar plot shows the marginal and common effects of these components. Constrained and residual variations are 86.5% and 13.5%, respectively.

HP and VP revealed that environmental distances (individual effect 80.54%, *p* < 0.001, Figure 7) predominated over geographical distances (individual effect 9.95%, *p* > 0.05, Figure 7), collectively explaining 87.9% of the variance in structural indices (Figure 7). This strong predominance of environmental distance in explaining structural variation aligns with the stage‐specific shifts in functional and phylogenetic structures (Figure 6) and the higher explanatory power of environmental versus geographical distance. These results further support the dominant role of environmental filtering in community assembly across successional stages. In community ecology, geographical distance is commonly considered a proxy for spatial processes associated with dispersal limitation. However, given its low and non‐significant independent effect, its contribution to community assembly in this study system appears limited. Notably, environmental and geographical distances exhibited marginal effects of 77.97% and 7.38%, respectively, with a positive common effect (2.57%) observed between them (Figure 7), indicating that environmental variation is partly spatially structured.

## 4 Discussion

Based on functional trait and phylogenetic analyses of community assembly, it is commonly reported that species with close phylogenetic relationships exhibit similar functional traits (Webb et al. 2003). However, previous studies indicate that phylogeny is insufficient to explain all functional trait information, as not all functional traits exhibit significant phylogenetic signals (Swenson 2013). Consequently, relying solely on functional traits or phylogeny to interpret community assembly mechanisms is inherently constrained (Flynn et al. 2011).

Our results revealed that functional traits were significantly influenced by phylogeny, though this conservatism followed a dynamic unimodal pattern across successional stages rather than a static one. Conservatism was suppressed in Stages I–II, peaked in Stage III (*K* > 1 for NFT, PM, and SDM; *p* ≤ 0.05, Figure 4), and attenuated thereafter. This suggests assembly mechanisms shift fundamentally during succession (Letcher et al. 2012). In early stages, severe abiotic stress in karst habitats imposes strong environmental filtering, selecting for convergent traits and depressing K values (Purschke et al. 2013). By mid‐succession (Stage III), niche conservatism emerges as the primary driver for conserved structural traits, allowing relatives to co‐occur. However, intensified competitive exclusion overrides this effect in later stages, as canopy closure drives trait divergence among co‐occurring species (Lasky et al. 2014). This shift from filtering to competition highlights a distinct threshold response in community assembly, aligning with classical trajectories observed in non‐karst successions (Lasky et al. 2014; Poorter et al. 2021). However, unlike non‐karst forests, where functional shifts are often linear and driven by simple resource acquisition trade‐offs (Lohbeck et al. 2013), the unique lithological constraints of karst systems prolong edaphic stress. This delayed transition uniquely shapes the unimodal peak of trait conservatism before canopy‐driven divergence dominates. These dynamic shifts directly address the phylogenetic patterns underlying community diversity.

In this study, nine indices of taxonomic, functional, and phylogenetic *α* diversity were selected to represent plant diversity dynamics at different successional stages (Figure 5). Overall, the changes in taxonomic, functional, and phylogenetic *α* diversity were not uniform. This finding is consistent with observations from different successional stages of the Brazilian rainforest (Romanowski et al. 2021), as well as classical successional trajectories in non‐karst temperate regions (Purschke et al. 2013). There were certain correlations among the three facets of taxonomic, functional, and phylogenetic *α* diversity, but their changes were not exactly synchronous. This may be due to the special habitats and rich biodiversity in these distinct regions. While environmental filtering and competitive exclusion drive this decoupling across most ecosystems (Lasky et al. 2014), the temporal dynamics of these assembly rules in karst habitats are unique. In typical non‐karst secondary succession, rapid soil development gradually relaxes abiotic filters, shifting the primary assembly driver toward biotic competition (Poorter et al. 2021). In contrast, the severe edaphic constraints of karst ecosystems impose a prolonged and dominant environmental filter. Consequently, this persistent abiotic stress strongly regulates functional and phylogenetic convergence even as taxonomic diversity recovers, distinguishing the karst assembly rules from classical non‐karst models.

The identification of functional traits and the detection of phylogenetic structures in a community are based on distinct sets of information, and the phylogenetic signals of functional traits signify the associations between functional traits and phylogeny (Kraft et al. 2007). Our results demonstrated significant phylogenetic signals in most functional traits, indicating strong phylogenetic conservatism (Figure 4). Concurrently, as succession progressed, the NRI showed overdispersion in stage VI and clustering in the other six stages. The traitMPD, traitMNTD, and NTI showed overdispersion during stages I, II, and III, followed by clustering in stages IV, V, VI, and VII (Figure 6). This temporal shift indicates that limiting similarity initially dominated, where co‐occurring species were distantly related to minimize niche overlap (Webb et al. 2003). However, as succession advanced, the severe lithological constraints and shallow soils characteristic of karst systems prolonged the influence of habitat filtering (Lohbeck et al. 2013). Unlike typical forests where filters relax as soils develop (Poorter et al. 2021), the increasing scarcity of nutrients in karst habitats intensifies environmental stress, selectively favoring conserved stress‐tolerant clades. Consequently, the assembly mechanism evolves from competition‐driven divergence to stress‐driven convergence. This site‐specific trajectory contrasts with non‐karst models where stochastic processes or negative density dependence often dominate mature stages (Lebrija‐Trejos et al. 2010), highlighting that karst assembly is defined by a re‐emergence of filtering dictated by habitat harshness.

Traditional successional theories often suggest a trajectory from clustering to overdispersion, as competitive exclusion typically becomes more pronounced in stable, late‐successional environments (Whitfeld et al. 2012; Han et al. 2023). However, our findings align with the habitat filtering hypothesis, where severe environmental stressors act as a persistent sieve. In the karst evergreen deciduous broad‐leaved forest, the unique edaphic constraints impose stringent physiological filters. Unlike mesic non‐karst forests where biotic interactions often dampen environmental signals over time (Lasky et al. 2014), the ecologically fragile nature of karst systems forces a sustained reliance on niche‐based filtering. This is consistent with observations in other abiotic‐stress‐dominated systems, including subalpine meadows and the Loess Plateau (Liu et al. 2021; Chai et al. 2023), highlighting that habitat harshness can override the divergent effects of competition during forest maturation.

Community assembly is governed by the interplay between deterministic niche processes and stochastic neutral processes. Our results demonstrate that niche‐based habitat filtering predominantly shapes community assembly across karst successional stages, with environmental distance explaining 80.54% of the variation (*p* < 0.001, Figure 7), whereas the effect of dispersal limitation remained non‐significant (9.95%, *p* > 0.05, Figure 7). This stark dominance of environmental selection over stochasticity contrasts with many tropical lowland forests, where dispersal limitation often plays a more substantial role due to higher biological connectivity and more homogeneous substrates (Hubbell 2001; Condit et al. 2002). In contrast, the extreme environmental filtering, characterized by shallow soils and severe water‐alkali stress in karst systems, mirrors the assembly patterns found in high‐latitude alpine or arid ecosystems, where abiotic constraints override neutral dynamics (Chase 2007; Graham et al. 2010). These findings suggest that while neutrality may emerge in productive, resource‐rich forests, the environmental harshness of karst habitats strengthens niche partitioning, aligning with the continuum hypothesis, which posits that the relative importance of assembly processes shifts along stress gradients (Gravel et al. 2006).

These mechanistic insights provide a species‐selection recipe for karst restoration (Laughlin 2014). In early succession (Stages I–III), the observed overdispersion suggests that interspecific competition dominates. To accelerate soil C and N sequestration, managers should prioritize pioneer species that are nitrogen‐fixing and deciduous, such as *Albizia kalkora* and *Biancaea decapetala*. Conversely, the clustering in later stages (Stages IV–VII) reflects intensified environmental filtering. To boost phylogenetic diversity and drought insurance, late‐successional portfolios should include phylogenetically distinct stress‐tolerant species like *Handeliodendron bodinieri*, *Fraxinus insularis*, and *Platycarya strobilacea* , which balance resource‐use efficiency with high karst adaptation.

Despite these implications, certain limitations warrant consideration. First, the spatial scale of this study was restricted to a specific karst chronosequence; future work should employ multi‐site comparisons to validate the unimodal pattern of trait conservatism across broader environmental gradients. Second, while nine traits were analyzed, the exclusion of intraspecific trait variation may underestimate the role of local adaptation in community assembly. Finally, although edaphic factors explained 80.54% of the variation, integrating below‐ground processes could provide a more holistic understanding of how karst plants navigate lithological constraints.

## 5 Conclusions

The community assembly mechanisms of woody plants at various successional stages in the studied karst region were systematically investigated using the space‐for‐time substitution approach, based on functional traits and phylogeny. The findings revealed significant but dynamically unimodal phylogenetic conservation of the functional traits in the area. During the initial phase of succession, intense interspecific competition drove trait divergence, and community assembly was influenced primarily by biotic interactions. However, in later stages, severe edaphic constraints led to a shift in the dominant ecological process toward habitat filtering and species convergence. Furthermore, niche processes driven by environmental distance dominated community assembly, while the impact of neutral processes associated with geographical distance was negligible. These findings highlight the need for stage‐specific species selection in karst ecosystems to facilitate the restoration and reconstruction of karst plant communities.

## Author Contributions

**Hongfen Hu:** conceptualization (equal), data curation (equal), software (equal), visualization (equal), writing – original draft (equal). **Mingtai An:** conceptualization (equal), investigation (equal), methodology (equal), supervision (equal), writing – review and editing (equal). **Kun Wang:** data curation (equal), software (equal), visualization (equal). **Li Tian:** data curation (equal), methodology (equal), visualization (equal). **Feng Liu:** methodology (equal), software (equal), visualization (equal). **Yiran Wang:** data curation (equal).

## Funding

This work was supported by the National Natural Science Foundation of China (NSFC) under Grant No. 32560328 (Project title: Key Environmental Factors for the Formation of Small‐Scale Vertical Zonation of Forest Vegetation in the Maolan Karst Peak‐Clusters); the Special Survey on Resources and Survival Status of Characteristic Coniferous Forest on Summits of Karst Peak‐Clusters in Maolan Nature Reserve (Phase I) (Grant No. DXZY‐2025‐CG‐027); and the Maolan Forest Dynamics Plot 2024 Annual Monitoring (GZZH‐2024‐ZB228).

## Conflicts of Interest

The authors declare no conflicts of interest.

## Supporting information


**Data S1.** 3 Phylogenetic tree files.

**Data S2.** Environmental data, diversity and structural indices.

**Data S3.** Functional traits.

## Data Availability

All data and code supporting the conclusions of this manuscript are available in ScienceDB at the permanent link: (https://www.scidb.cn/s/Q7vqUn). Supporting Information related to the study is also included in the manuscript submission.
