# Supplementary material for: Community Assembly Mechanism of Woody Plants at Different Successional Stages in Karst Areas Based on Functional Traits and Phylogeny
Source: Ecol Evol. 2026 May 18;16(5):e73661. doi: 10.1002/ece3.73661 (PMC13183475; doi:10.1002/ece3.73661)
Supplement: Supplementary file 1 — Data S1. 3 Phylogenetic tree files. [file ECE3-16-e73661-s001.docx]

((((((((((((((((((Dendropanax_hainanensis:13.02075,Kalopanax_septemlobus:13.020751):0.40374,Brassaiopsis_glomerulata:13.424491):11.129759,Aralia_elata:24.55425):38.814949,(Pittosporum_trigonocarpum:15.319821,Pittosporum_brevicalyx:15.319821,((Pittosporum_illicioides:2.700261,Pittosporum_glabratum:2.700261):0.670544,Pittosporum_tobira:3.370805):11.949017):48.049377):22.271456,(((Zabelia_biflora:1.970545,Zabelia_dielsii:1.970546):48.781128,Lonicera_japonica:50.751674):20.186291,(Viburnum_propinquum:14.931916,Viburnum_henryi:14.931916):56.006049)Dipsacales.rn.d8s.tre:14.70269)mrcaott1673ott2128:17.052186,((Ilex_macrocarpa:43.189173,Ilex_chinensis:43.189173):48.784741,Gomphandra_tetrandra:91.973914)Aquifoliales.rn.d8s.tre:10.718927)campanulids:4.04857,((((((((Clerodendrum_mandarinorum:7.887564,Clerodendrum_cyrtophyllum:7.887565):21.488324,Premna_puberula:29.375888):0.906324,Callicarpa_bodinieri:30.282212):0.32735,Vitex_negundo:30.609563):18.263848,Buddleja_officinalis:48.873411):22.147198,(Chengiodendron_matsumuranum:32.094045,(((Chionanthus_ramiflorus:17.739301,Fraxinus_insularis:17.7393):2.37228,(Ligustrum_sinense_var._myrianthum:2.538181,Ligustrum_sinense:2.538182):17.573399):9.878158,(Jasminum_seguinii:20.618526,Jasminum_lanceolaria:20.618526,Jasminum_nervosum:20.618527):9.371212):2.104307):38.926563):18.730459,((((Lasianthus_chinensis:46.572028,(Coptosapelta_diffusa:40.311829,Luculia_pinceana:40.311829):6.260199):3.177481,((((Tarennoidea_wallichii:15.190966,Ixora_henryi:15.190965):7.945043,Mussaenda_pubescens:23.136009):0.731303,Emmenopterys_henryi:23.867312):4.157811,(Sinoadina_racemosa:4.140018,Metadina_trichotoma:4.140018):23.885105):21.724386):17.978354,((Periploca_calophylla:21.894458,((Trachelospermum_jasminoides:0.800378,Trachelospermum_axillare:0.800378):12.402691,Cleghornia_malaccensis:13.203069):8.69139):9.000046,(Rauvolfia_verticillata:25.442097,Alyxia_sinensis:25.442097):5.452408):36.833358)Gentianales.rn.d8s.tre:18.185274,(Lycium_chinense:66.649825,Tridynamia_sinensis:66.649824):19.263312)mrcaott1191ott2192:3.837931)mrcaott248ott1191:12.636506,(Mappianthus_iodoides:69.350537,Aucuba_chinensis:69.350537)mrcaott100191ott148281:33.037037)lamiids:4.353837)mrcaott248ott320:5.599318,((Actinidia_callosa_var._henryi:93.233447,((Symplocos_adenophylla:3.472871,Symplocos_sumuntia:3.472871):77.712363,Styrax_japonicus:81.185234):12.048214):1.649845,((((((Ardisia_faberi:10.217012,Ardisia_crenata:10.217013):2.003968,Embelia_laeta:12.220981):1.315892,(Myrsine_semiserrata:5.576693,Myrsine_seguinii:5.576694):7.960179):47.188082,(Maesa_perlarius:1.952241,Maesa_japonica:1.952242):58.772713):26.229643,(Diospyros_kaki_var._silvestris:10.08508,Diospyros_dumetorum:10.08508,Diospyros_kaki:10.085081):76.869517):7.403593,(Sinosideroxylon_pedunculatum:61.911798,Camellia_costei:61.911799):32.446392):0.525102):17.457436)mrcaott248ott650:2.225571,(Hydrangea_strigosa:97.987565,((Cornus_elliptica:47.710426,Cornus_wilsoniana:47.710427):20.298601,Alangium_faberi:68.009027):29.978537):16.578735)mrcaott248ott27233:6.818365,Schoepfia_jasminodora:121.384665)mrcaott248ott19688:2.349572,(((((((Puhuaea_sequax:84.763337,Platyosprion_platycarpum:84.763337,Ohwia_caudata:84.763337,Cheniella_glauca:84.763337,Biancaea_decapetala:84.763337,(((Callerya_nitida:57.631492,(Lespedeza_thunbergii_subsp._elliptica:44.053628,Derris_taiwaniana:44.053628):13.577863):6.584434,((Dalbergia_hancei:58.86651,Ormosia_henryi:58.86651):0.333846,Styphnolobium_japonicum:59.200356):5.01557):13.747261,((((Albizia_kalkora:17.529367,Archidendron_clypearia:17.529367):26.043174,Erythrophleum_fordii:43.572541):4.254917,Pterolobium_punctatum:47.827457):10.275458,Gleditsia_sinensis:58.102916):19.860271):6.800151,Lysidice_rhodostegia:84.763338):11.311575,Polygala_caudata:96.074912):16.626283,((((((Rosa_laevigata:49.062754,Rosa_cymosa:49.062754):6.528052,Rosa_rubus:55.590806):29.411982,(Rubus_xanthoneurus:84.30543,(Rubus_innominatus:35.599657,Rubus_niveus:35.599657):48.705774):0.697357):9.558392,((Spiraea_chinensis:49.606202,(((Photinia_glabra:8.347551,Photinia_bodinieri:8.347552,Photinia_parvifolia:8.347552):1.267247,(Pyracantha_loureiroi:2.67247,Pyracantha_fortuneana:2.672471):6.942328):1.130995,(Eriobotrya_cavaleriei:8.379749,Rhaphiolepis_indica:8.379749):2.366045):38.860409):1.127065,(Prunus_pseudocerasus:28.162008,Prunus_zippeliana:28.162008):22.57126):43.827912):4.400616,((((((((Ficus_sarmentosa_var._henryi:11.922425,Ficus_irisana:11.922425,((Ficus_heteromorpha:0.560549,Ficus_erecta:0.560549):3.895787,Ficus_hirta:4.456336):7.46609):28.104134,(Broussonetia_monoica:17.419782,Broussonetia_kaempferi:17.419783):22.606777):1.729536,Maclura_cochinchinensis:41.756096):0.707801,Morus_wittiorum:42.463897):26.048553,(((Debregeasia_longifolia:2.332891,Debregeasia_orientalis:2.332891):27.921686,Pouzolzia_sanguinea:30.254576):4.838348,Oreocnide_frutescens:35.092925):33.419525):4.956685,(Pteroceltis_tatarinowii:67.169797,Celtis_cerasifera:67.169796):6.299338):5.701507,(Ulmus_parvifolia:8.915005,Zelkova_serrata:8.915005):70.255637):6.322774,((Paliurus_ramosissimus:51.398491,Hovenia_acerba:51.398492,(((Rhamnus_esquirolii:7.134798,Rhamnus_dumetorum:7.134798):3.799781,Frangula_crenata:10.93458):13.353658,(((Rhamnella_rubrinervis:17.368875,Rhamnella_martini:17.368875):4.608188,Berchemia_sinica:21.977063):1.183798,(Sageretia_rugosa:9.260566,Sageretia_hamosa:9.260567,Sageretia_thea:9.260567):13.900295):1.127376):27.110254):24.21016,(Elaeagnus_pungens:10.517553,Elaeagnus_lanceolata:10.517553,Elaeagnus_glabra:10.517553):65.091098):9.884764):13.46838)Rosales.rn.d8s.tre:12.186212,(Coriaria_nepalensis:109.068485,((((Carpinus_kweichowensis:9.925305,(Carpinus_viminea:8.226376,Carpinus_pubescens:8.226376):1.69893):79.832858,(Morella_rubra:12.187724,Morella_esculenta:12.187725):77.570439):0.890866,(Platycarya_strobilacea:50.201096,Engelhardia_roxburghiana:50.201096):40.447933):7.230533,((Quercus_shennongii:11.776698,Quercus_jenseniana:11.776698,((Quercus_myrsinifolia:2.418418,Quercus_glauca:2.418418):5.190545,Quercus_multinervis:7.608963):4.167736):0.532457,(Castanopsis_fargesii:9.977144,(Lithocarpus_harlandii:7.383389,Lithocarpus_confinis:7.383389,(Lithocarpus_hancei:3.203055,(Lithocarpus_henryi:2.543091,Lithocarpus_glaber:2.543091):0.659964):4.180335):2.593754):2.332012):85.570407):11.188922)mrcaott2511ott32687:2.079523)mrcaott371ott2511:1.553188)mrcaott371ott579:3.084369,(((((Carrierea_calycina:62.60732,(Populus_adenopoda:46.049138,((Xylosma_controversa:13.697703,(Xylosma_congesta:7.560352,Xylosma_longifolia:7.560352):6.137352):21.546755,(Itoa_orientalis:23.222644,Bennettiodendron_leprosipes:23.222644):12.021815):10.804679):16.558183):32.304265,(Phyllanthodendron_dunnianum:71.925139,(Glochidion_puberum:54.25068,Leptopus_chinensis:54.25068):17.67446):22.986446):7.696173,((((Triadica_rotundifolia:3.436638003,Triadica_sebifera:3.436638003):48.434216,Vernicia_fordii:51.870854):10.025653,(Mallotus_tenuifolius:8.527074003,Mallotus_barbatus:8.527075003,(Mallotus_repandus:5.813106003,Mallotus_philippensis:5.813106003):2.713969):53.369432):32.31013,Tirpitzia_sinensis:94.206637):8.401122):8.818668,(Elaeocarpus_japonicus:64.13408,Sloanea_sinensis:64.13408):47.292347)mrcaott2ott345:0.594159,((Salacia_sessiliflora:33.526443,(((Euonymus_nitidus:6.299247,Euonymus_dielsianus:6.299247):4.297401,Euonymus_acanthocarpus:10.596648):14.201731,(Celastrus_stylosus:3.301217,((Celastrus_gemmatus:0.715341,Celastrus_orbiculatus:0.715341):1.056532,Celastrus_hindsii:1.771873):1.529344):21.497162):8.728065):36.636814,Microtropis_triflora:70.163258):41.857328)mrcaott2ott1479:3.764979)mrcaott2ott371:2.793039,((((((Urena_lobata:25.543721,Firmiana_simplex:25.543721):7.766866,Reevesia_pubescens:33.310586):36.807499,Wikstroemia_micrantha:70.118085):34.139973,((((((((Zanthoxylum_dissitum:27.931214,(Zanthoxylum_armatum:20.312474,Zanthoxylum_scandens:20.312474):7.618741):1.580378,Toddalia_asiatica:29.511593):8.839498,(Phellodendron_sinii:2.724522,Phellodendron_chinense_var._glabriusculum:2.724522):35.626568):8.317546,(Tetradium_ruticarpum:7.250762,Tetradium_glabrifolium:7.250762):39.417875):7.710079,((Clausena_dunniana:16.876309,Clausena_lansium:16.87631):9.910894,Murraya_exotica:26.787203):27.591512):14.90101,(Toona_sinensis:66.889798,Picrasma_quassioides:66.889798):2.389928):9.868676,(Boniodendron_minius:60.766221,(((Acer_wangchii:14.095456,Acer_sycopseoides:14.095456,Acer_sinense:14.095456,Acer_cordatum:14.095456,Acer_fabri:14.095457):31.041674,Handeliodendron_bodinieri:45.137131):12.444139,(Koelreuteria_paniculata:23.974589,Koelreuteria_bipinnata:23.974589):33.606681):3.184952):18.38218):0.769939,(((Rhus_chinensis:17.702776,Pistacia_chinensis:17.702776):1.777426,(Toxicodendron_vernicifluum:8.617165,(Toxicodendron_succedaneum:1.129833,Toxicodendron_sylvestre:1.129833):7.487332):10.863037):23.87323,Choerospondias_axillaris:43.353432):36.564909):24.339718)mrcaott96ott378:8.559102,((Turpinia_affinis:5.829145,Turpinia_montana:5.829146):25.457578,Stachyurus_chinensis:31.286724):81.530437)mrcaott96ott14140:4.176035,((Oxyspora_paniculata:44.240951,Melastoma_candidum:44.240951):48.023447,Decaspermum_gracilentum:92.264399):24.728797)mrcaott96ott607:1.585408)mrcaott2ott96:2.70865,((Ampelopsis_glandulosa:46.736734,Nekemias_chaffanjonii:46.736733):5.387923,((Vitis_flexuosa:27.698941,((Vitis_davidii:19.553555,Vitis_bellula:19.553555):1.447807,Vitis_heyneana:21.001362):6.697579):22.131672,(Parthenocissus_dalzielii:23.125136,Parthenocissus_semicordata:23.125136):26.705477):2.294044):69.162597)mrcaott2ott8384:1.117838,(Distylium_myricoides:94.1210646,(Liquidambar_formosana:92.665891,Daphniphyllum_calycinum:92.665891):1.4551736):28.2840274)mrcaott2ott2464:1.329145)Pentapetalae:4.81969,Sarcococca_ruscifolia:128.553926)mrcaott2ott8379:1.770604,(Meliosma_rigida:68.191568,Sabia_swinhoei:68.191568):62.132963)mrcaott2ott969:1.356169,((((Clematis_finetiana:4.058817,Clematis_uncinata:4.058817):76.22424,(Mahonia_fortunei:42.942157,Nandina_domestica:42.942157):37.3409):9.642483,Cocculus_laurifolius:89.92554):8.272723,(Holboellia_latifolia:10.605102,Akebia_trifoliata:10.605102):87.593161):33.482437)eudicotyledons:4.077365,(((Hsuehochloa_calcarea:47.740111,(Bonia_saxatilis:23.962961,(Chimonobambusa_angustifolia:1.548546,Phyllostachys_heteroclada:1.548545):22.414416):23.77715):60.495041,Rhapis_excelsa:108.235153)mrcaott121ott252:8.641654,((Smilax_japonica:9.374078,(Smilax_china:3.654699,Smilax_glaucochina:3.654699):5.71938):0.534029,Smilax_microphylla:9.908108):106.968699)mrcaott121ott1439:18.881258)mrcaott2ott121:0.154122,(((((Fissistigma_polyanthum:10.27346,Fissistigma_polyanthoides:10.27346):36.438822,Artabotrys_hongkongensis:46.712281):8.784217,Miliusa_balansae:55.496499):52.883603,(Woonyoungia_septentrionalis:65.271435,Lirianthe_championii:65.271435,Houpoea_officinalis:65.271435,Michelia_martini:65.271435):43.108666):19.321085,(((((Cinnamomum_parthenoxylon:8.762164,(Cinnamomum_wilsonii:7.95558,Cinnamomum_camphora:7.95558):0.806585):1.25665,(((Litsea_elongata_var._faberi:9.419435,Litsea_coreana_var._sinensis:9.419435,Litsea_elongata:9.419436,Litsea_pungens:9.419436):0.055318,(Neolitsea_aurata:4.211674,Neolitsea_pinninervis:4.211674):5.26308):0.219654,(Lindera_pulcherrima_var._hemsleyana:9.172666,Lindera_megaphylla:9.172666,(Lindera_communis:6.315462,Lindera_glauca:6.315462):2.857205):0.521741):0.324407):15.234767,((Machilus_chuanchienensis:5.538614,(Machilus_microcarpa:1.552891,Machilus_ichangensis:1.552891):3.985724):12.42638,(Phoebe_hui:7.282083,Phoebe_crassipedicella:7.282083):10.682911):7.288587):55.552625,Beilschmiedia_kweichowensis:80.806207):24.288042,Illigera_parviflora:105.094248):22.606938)mrcaott890ott9684:8.211)Mesangiospermae:1.781936,Schisandra_chinensis:137.694123)mrcaott2ott35778:187.355905,Cephalotaxus_fortunei:325.050028)Spermatophyta;
